# Laparoscopic Lymph Node Assessment Prior to Open Radical Hysterectomy—A Two-Center Retrospective Study on Individualized Surgical Management of Early-Stage Cervical Cancer

**DOI:** 10.3390/jpm16090475

**Published:** 2026-09-15

**Authors:** Magdalena Postl, Christoph Grimm, Melina Danisch, Patrik Petrov, Mara Mantovan, Alexander Reinthaller, Eva Langthaler, Stephan Polterauer, Christian Marth, Nicole Concin, Alain Gustave Zeimet

**Affiliations:** 1 Department of Obstetrics and Gynecology, Division of General Gynecology and Gynecological Oncology, Comprehensive Cancer Center, Gynecologic Cancer Unit, Medical University of Vienna, 1090 Vienna, Austria; magdalena.postl@meduniwien.ac.at (M.P.);; 2 Department of Obstetrics and Gynecology, Medical University Innsbruck, 6020 Innsbruck, Austria; 3 Department of Pathology, Medical University Vienna, 1090 Vienna, Austria

**Keywords:** uterine cervical neoplasms, early-stage cervical cancer, radical hysterectomy, laparoscopy, surgical staging, lymphatic metastasis, laparotomy, progression-free survival, chemoradiotherapy

## Abstract

**Background/Objectives**: Recent evidence suggests open radical hysterectomy is safer than radical hysterectomy by minimally invasive surgery (MIS) in early-stage cervical cancer. This study evaluated the oncological safety of laparoscopic lymph node assessment followed by open radical hysterectomy and the rate of unnecessary laparotomies avoided compared with a completely open approach. **Methods**: This two-center retrospective study included patients with cervical cancer FIGO stage ≤ Ib3 treated at two Austrian centers between 2015 and 2023. Patients undergoing laparoscopic lymph node assessment followed by open radical hysterectomy were compared with a matched historical cohort treated with primary open surgery between 2012 and 2017. Recurrence rates and progression-free survival (PFS) were compared using univariate and multivariate analyses. **Results**: In total, 130 patients were included: 79 (60.8%) underwent the combined approach and 51 (39.2%) the completely open approach. Recurrence rates (10.1% vs. 5.9%, *p* = 0.53) and median PFS (*p* = 0.36; median 26.0 vs. 71.3 months) did not differ between the two approaches. All patients underwent thorough preoperative radiologic and clinical assessment without suspected nodal metastases and were scheduled for radical hysterectomy with prior nodal assessment by MIS or open surgery. Intraoperative lymph node metastases were incidentally detected in 21/130 (16.2%) patients (combined approach *n* = 12 [15.2%] vs. open approach *n* = 9 [17.6%]). In cases of positive lymph nodes, surgery was abandoned without performing open radical hysterectomy and patients were referred to chemoradiotherapy. The needed to treat number to prevent one unnecessary open surgery was 6.25. **Conclusions**: Laparoscopic lymph node assessment before open radical hysterectomy appears oncologically safe, with comparable recurrence rates and PFS, and could spare one unnecessary laparotomy in every 7th patient with early-stage cervical cancer undergoing the combined approach.

## 1. Introduction

In early-stage cervical cancer, radical hysterectomy was traditionally performed via an open or vaginal approach. Starting in the early 1990s, minimally invasive techniques, either by conventional laparoscopic or robotically assisted laparoscopic surgery, were increasingly implemented [1,2,3]. Multiple retrospective studies highlighted the clinical effectiveness and oncological safety of laparoscopic radical hysterectomy [4,5,6,7]. However, in 2018, the “Laparoscopic Approach to Cervical Cancer—LACC” trial, a prospective multicenter study including over 600 patients, reported that minimal invasive radical hysterectomy was associated with impaired disease-free and overall survival (OS) and with a higher rate of locoregional recurrence compared to open radical hysterectomy [8]. The unexpected results of the LACC trial led to a paradigm change and open radical hysterectomy is again considered the preferred surgical treatment approach in early-stage cervical cancer [9].

A clinical aspect, which was not examined in the LACC trial, is the management of lymph node evaluation. As recommended by the European Society of Gynecological Oncology (ESGO) in their guidelines [10,11], intraoperative lymph node staging should be performed as the first step during surgical management. If positive lymph nodes are detected, further surgical steps, including systematic pelvic lymph node dissection and radical hysterectomy, should be abandoned and patients should be referred to primary chemoradiotherapy (CRT) [10,11]. In early-stage cervical cancer, 17–19% of patients are diagnosed with metastatic lymph nodes at surgery. Transferring all early-stage cervical cancer patients directly to open surgery—in line with the LACC trial—would thus lead to 17–19% of patients undergoing unnecessary open surgery [12,13].

One potential approach to address this challenge is to first assess the lymph node status by MIS and, in case of negative lymph nodes—either on a frozen section or final pathology—continue to perform a subsequent open radical hysterectomy. In cases of pathologically confirmed lymph node metastases, patients can be spared unnecessary open surgery and be directly referred to primary CRT.

The aim of this study was to assess the oncological safety of the combined approach by comparing recurrence rates and progression-free survival (PFS) in patients with early-stage cervical cancer, who underwent laparoscopic lymph node assessment followed by open radical hysterectomy (combined approach) compared to a historic control group with a completely open approach for both lymph node assessment and radical hysterectomy. Furthermore, we aimed to define the number needed to treat (NNT) to spare one unnecessary laparotomy within the cohort of patients undergoing the combined approach.

## 2. Materials and Methods

### 2.1. Patients

This retrospective study included all patients who were preoperatively diagnosed with early-stage cervical cancer (FIGO stage ≤ Ib3, as preoperatively assumed) and underwent laparoscopic lymph node assessment prior to open radical hysterectomy as primary treatment at the Medical Universities of Vienna and Innsbruck between 2015 and 2023. To compare the data with a matched historical group, we further included patients with early-stage cervical cancer who underwent completely open surgery at the Medical University of Vienna between 2012 and 2017. Patients’ clinical data were collected from available tumor databases and by review of electronic charts.

Tumor staging was performed according to the 2018 International Federation of Gynecology and Obstetrics (FIGO) classification system [14]. Early-stage cervical cancer was defined as FIGO stage ≤ Ib3. Cervical cancer was histologically diagnosed either by biopsy or by cervical conization. Tumor assessment was performed by imaging and by clinical examination. Imaging included a computed tomography (CT) scan of the chest, abdomen, and pelvis and magnetic resonance imaging of the pelvis. Since 2018, a CT scan has typically been performed as a positron emission tomography–computed tomography (PET-CT) scan. Treatment was based on local standards and international guidelines. In the study group (combined approach), the surgical approach consisted of laparoscopic systematic lymphadenectomy or sentinel lymph node (SLN) biopsy followed by open radical hysterectomy in the case of negative lymph nodes assessed either by frozen section or by final pathology. Ultrastaging was routinely performed to confirm the absence of lymph node metastases. Open radical hysterectomy was generally performed via Pfannenstiel incision. If metastases were found in the pelvic lymph nodes, radical hysterectomy was abandoned. In the majority of cases with pelvic lymph node metastases, surgical para-aortic lymph node assessment by MIS was carried out. These patients received primary CRT. In the control group (completely open approach), laparotomy with systematic lymphadenectomy or SLN biopsy followed by radical hysterectomy in the case of negative lymph nodes was performed. In selected high-risk cases, adjuvant CRT after open radical hysterectomy was also administered to patients with negative lymph nodes but adverse pathological features, such as tumor size ≥ 4 cm and additional risk factors such as deep stromal invasion and/or lymphovascular space invasion.

All patients were included in the institutions’ 10-year follow-up program, which includes clinical examination, Papanicolaou (PAP) smear, human papillomavirus (HPV) test, and regular radiologic assessments. All patients were seen four times annually for three years, twice per year in the following three years, and once annually for an additional four years. If recurrence was assumed, biopsy was taken, and radiologic assessment was performed if required. Radiologic assessment was performed once a year and, in addition, whenever clinically indicated, i.e., in the case of symptoms or suspicious findings on clinical examination, PAP smear or HPV testing. Imaging consisted of a CT of the chest, abdomen and pelvis, PET-CT and/or MRI of the pelvis at the discretion of the treating physician.

### 2.2. Ethics

Prior to initiation, this study was approved by the Institutional Review Board (IRB) of the Medical University of Vienna (IRB approval number: 1551/2021) and Innsbruck Medical University (ECS 1438/2021). All patients gave consent to treatment according to institutional guidelines and to the pseudonymized assessment of clinical data and treatment outcome. As this was a retrospective trial, the Institutional Review Board waived the requirement to obtain distinct written informed consent from each individual patient. Patients’ data were pseudonymized and de-identified prior to analysis. The study was performed according to the Declaration of Helsinki, the ICH Harmonized Tripartite Guideline for Good Clinical Practice, and the guidelines of the Institutional Review Board of the Medical Universities of Vienna and Innsbruck.

### 2.3. Statistical Analysis

Data are presented as medians with interquartile ranges (IQRs) for continuous variables and as absolute frequencies and percentages for categorical variables. The two surgical approaches were compared regarding demographic and clinical characteristics using the Mann–Whitney U test for continuous variables, and the Pearson chi-squared test or Fisher’s exact test for categorical variables. All statistical tests were two-sided, and a *p*-value < 0.05 was considered statistically significant.

PFS was defined as the time from initial diagnosis to the first documented recurrence and served as the primary endpoint. OS was defined as the time from diagnosis to death or last follow-up, with survivors censored at the last date they were known to be alive. Since only one disease-specific death was observed in the completely open approach group, further OS analysis was not performed due to a lack of events. Therefore, only PFS was compared between groups using the log-rank test. Univariate and multivariate Cox proportional hazards regression analyses were performed to assess the association between clinical and pathological variables including patient age (≤65 vs. >65 years), histological type (squamous vs. other), FIGO tumor stage (FIGO Ia1–Ib1 vs. Ib2–IIIc2), and histological grade (G1 vs. G2 + G3) and PFS. Hazard ratios (HRs) were not reported due to the limited number of events (death and recurrence). Statistical analyses were performed using IBM SPSS Statistics, Version 30.0.0.0 for MAC (IBM Corp., Armonk, NY, USA).

## 3. Results

In total, 130 patients with early-stage cervical cancer and complete clinical information were included in the final analysis. In total, 79 patients (60.8%) underwent the combined approach and 51 patients (39.2%) underwent the completely open approach. No significant differences in demographic characteristics were observed between the groups, except for the prevalence of non-squamous cell carcinoma, which was significantly higher in the combined approach group (40.5%) compared to the completely open approach group (19.6%, *p* = 0.01). Patients’ characteristics are shown in Table 1.

Rates for SLN biopsy and systematic pelvic lymphadenectomy varied substantially between the combined group and the completely open group. This difference is due to the comparison of a recent combined group to a historic completely open group, when the SLN biopsy had not yet been widely implemented. Rates for SLN biopsy and systematic pelvic lymph node dissection were 84.8% and 15.2% in the combined approach group compared to 43.1% and 56.9% in the completely open approach group, respectively (*p* < 0.001).

The rates of adjuvant therapy were comparable between both groups. In total, 5/79 (6.3%) patients in the combined approach group and 6/51 (11.8%) patients in the completely open approach group received adjuvant CRT. In the combined approach, two patients (2.5%) had negative SLN in frozen section evaluation during surgery and micrometastases in the final pathologic evaluation after performance of ultrastaging; two patients (2.5%) were finally upstaged to FIGO stage IIb because pathologic parametrial infiltration was not detected on preoperative imaging and clinical assessment; and in one patient, (1.3%) the final pathology showed a tumor > 4 cm and substantial lymphovascular space invasion. In the completely open approach, 3/51 patients (5.9%) had negative SLN in frozen section evaluation during surgery and micrometastases at definitive pathologic diagnosis after performance of ultrastaging and three (5.9%) patients had large tumors (≥4 cm) and presence of additional risk factors.

In both groups, all patients underwent preoperative radiologic and clinical assessment without any hint of lymph node metastases and had therefore been scheduled for radical hysterectomy. In the combined approach group, 15.2% (*n* = 12/79) of patients had incidental intraoperative findings of lymph node metastases during MIS. These patients were directly referred to primary CRT without proceeding to open radical hysterectomy, thereby avoiding unnecessary laparotomy. In 12 out of 79 patients (15.2%) of the combined approach group, the two surgical steps were performed on separate dates, with open radical hysterectomy being scheduled only after final pathology had confirmed negative lymph nodes. In comparison, in the completely open approach group, open surgery had to be discontinued in 17.6% (*n* = 9/51) of patients due to incidental intraoperative diagnosis of lymph node metastases, resulting in unnecessary laparotomy in these patients. The NNT was 6.25 laparoscopic procedures to detect intraoperative lymph node metastases despite negative preoperative clinical and radiological findings to avoid one unnecessary laparotomy.

Of note, a comparison of a recent cohort (combined approach) with a historic cohort (completely open approach) leads to a substantial difference in median follow-up times between these two groups. Median follow-up time was 30 months in the combined approach group compared to 83 months in the open approach group (*p* = 0.19). Median PFS was 26.0 months in the combined approach group and 71.3 months in the completely open approach group (*p* = 0.36). Recurrences were observed in eight patients (10.1%) of the combined approach group and in three patients (5.9%) of the completely open approach group (*p* = 0.53). One disease-specific death (*n* = 1 [2%]) was observed in the completely open approach group.

**Figure 1:**
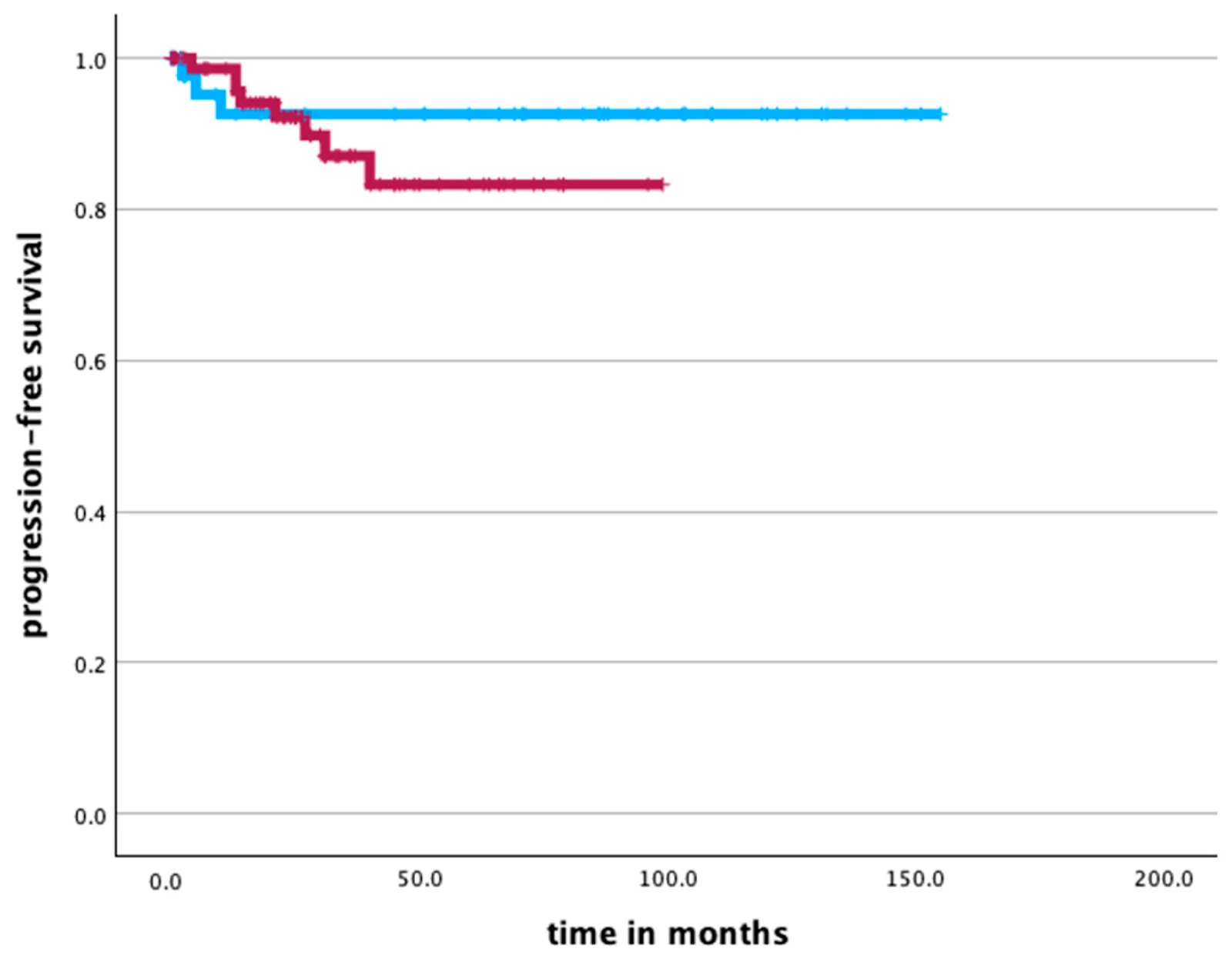
No significant difference in median progression-free survival between the combined and completely open approach was observed. The blue line depicts patients with cervical cancer FIGO ≤ Ib3 undergoing the completely open approach, and the red line depicts patients with cervical cancer FIGO ≤ Ib3 undergoing the combined surgical approach.

Univariate and multivariate analyses demonstrated that PFS was not significantly associated with patients’ age, histological type, FIGO stage, histological grade, and surgical approach (for details see Table 2). PFS for the combined approach and the completely open control is depicted through Kaplan–Meier curves in Figure 1.

## 4. Discussion

The present study revealed comparable oncological safety for a combined approach—comprising lymph node assessment by MIS followed by open radical hysterectomy—compared to a completely open approach in early-stage cervical cancer. Moreover, our study demonstrated an NNT of 6.25 for laparoscopic lymph node assessments prior to open radical hysterectomy to spare one laparotomy (based on 16.2% of patients with incidental intraoperative findings of lymph node metastases). With respect to PFS, the median PFS was 26.0 months in the combined approach group and 71.3 months in the completely open approach group (*p* = 0.36). This difference reflects the markedly different observation periods of a contemporary and a historical cohort rather than a difference in the course of the disease.

In the present study we did not observe a significant difference in recurrence rates and PFS between the two groups. Van de Lande et al. [15] reported surgical and oncological outcomes in a comparable number of patients undergoing the same combined surgical approach (*n* = 76) compared to a historical completely open approach (*n* = 93) cohort. In 13 patients (17.1%), surgery was discontinued after the lymph node assessment by MIS due to metastatic lymph nodes, which is in line with our findings. There was no difference in disease-free and disease-specific OS between the two groups. While Van de Lande et al. [15] analyzed data from 1988 to 2005 without SLN biopsy in the completely open approach and with only 56.6% in the combined approach, our study reflects contemporary FIGO 2018 staging and ESGO/ESMO (European Society For Medical Oncology) standards [10,11], with SLN biopsy and ultrastaging performed in 84.8% of patients in the combined approach group. Ultrastaging was not mentioned as part of the pathological assessment in the study by van de Lande et al. [15]. Michaan et al. [16] published their clinical experience on nine cases with the same combined surgical approach; however, this was done without providing information on follow-up and survival data. In two out of nine patients (22%), surgery was discontinued after the lymph node assessment by MIS due to metastatic lymph nodes. No false-negative cases in frozen section evaluation were reported [16].

The underlying reason for a worse oncologic outcome related to MIS in comparison with open surgery in cervical cancer, as demonstrated by the LACC trial [8] still needs to be clarified and is believed to be multifactorial. Recently, the MILACC study [17] re-analyzed the lymph nodes from patients with negative lymph nodes who subsequently recurred during the LACC trial. No low-volume lymph node metastases (micrometastases or isolated tumor cells) were found in any re-evaluated lymph nodes in either the MIS or the open surgical approach group. This finding argues against the hypothesis that the worse prognosis observed in the MIS arm results from endoscopic manipulation of lymph nodes harboring low-volume disease, leading to intraperitoneal tumor spread [17]. Nevertheless, the results of the LACC trial led to a worldwide switch in surgical strategy towards an open approach in the treatment of early-stage cervical cancer patients. Matsuo et al. showed a rapid decline in the rate of minimally invasive hysterectomy in the U.S., as patients were 63% less likely to be treated by MIS after the publication of the LACC trial [18]. Regarding postoperative complications or unplanned hospital readmissions, Schivardi et al. did not find a significant difference in the minimally invasive versus the open approach for radical hysterectomy. However, a higher rate of blood transfusion was observed in the open approach [19]. Of note, the LACC trial did not address the clinically relevant question of the surgical approach towards lymph node assessment, which is the recommended first surgical step in patients with early-stage cervical cancer according to ESGO/ESTRO (The European Society for Radiotherapy and Oncology)/ESP (European Society of Pathology) guidelines [10].

It is reported that lymph node metastases occur in up to 20% of patients with early-stage cervical cancer [12,13]. This aligns with the rates of 15.2% and 17.6% observed in our combined and completely open group, respectively. This group of patients is particularly important, as radical hysterectomy can be spared in this patient population. With the two-step MIS approach, unnecessary laparotomy can be avoided. With the broad implementation of ultrastaging as a standard pathological assessment of SLN, evidence on the incidence of isolated tumor cells and micrometastases is maturing. Therefore, a surgical two-step approach is becoming increasingly popular: initial MIS with pelvic SLN biopsy and pathological ultrastaging, followed by open radical hysterectomy in the case of negative SLN. According to our study, pelvic lymph node assessment could safely be performed by MIS, avoiding unnecessary laparotomy in patients with positive pelvic lymph nodes. These findings correspond to a calculated NNT of 6.25 to save one unnecessary open surgery.

Prospective multicenter studies with larger cohorts and extended follow-up, ideally randomized trials, are needed to confirm the oncologic safety of the combined approach. Further research should refine patient selection criteria and assess whether awaiting final ultrastaging results may be preferable to relying on intraoperative frozen section analysis, as well as evaluating perioperative morbidity, quality of life, and cost-effectiveness compared with the completely open approach.

To the best of our knowledge, this is the largest and most contemporary study presenting data on oncological safety of a combined surgical approach versus a completely open approach in patients with early-stage cervical cancer. Importantly, our study has several limitations. Due to the retrospective design and the historical control group, there are several imbalances concerning prognostic risk factors between the two study arms, such as the rate of non-squamous histologies and SLN biopsies. These prognostic risk factors were unevenly distributed between the two groups; however, no relevant associations with PFS were identified in univariate or multivariate analyses. It is very unlikely that the results reported herein are confounded by these differences.

In summary, the combined approach proved to be oncologically safe in the present study, with comparable recurrence rates and PFS. Approximately every seventh patient undergoing the combined approach can be spared an unnecessary laparotomy by a two-step surgical approach starting with lymph node assessment by MIS.

## Figures and Tables

**Table 1:** Patients’ characteristics stratified by surgical approach (combined surgical approach consisting of lymph node assessment by MIS followed by open radical hysterectomy versus completely open approach).

Parameter	N (%) or Median (IQR)		*p*-Value
	Combined Approach	Open Approach	
**Patients**			
	79 (60.8%)	51 (39.2%)	
**Patient’s age**			0.72 ^a^
	45.0 (37.0–55.0)	43.0 (36.0–54.3)	
**BMI**			0.15 ^a^
	23.9 (21.3–28.8)	22.7 (20.6–25.7)	
**ECOG**			0.52 ^b^
0	77 (97.5%)	51 (100.0%)	
1	2 (2.5%)	0	
**Histological type**			0.01 ^b^
Squamous	47 (59.5%)	41 (80.4%)	
Other	32 (40.5%)	10 (19.6%)	
**FIGO stage**			0.33 ^c^
IA1	3 (3.8%)	1 (2.0%)	
IA2	9 (11.4%)	2 (3.9%)	
IB1	23 (29.1%)	10 (19.6%)	
IB2	20 (25.3%)	18 (35.3%)	
IB3	8 (10.1%)	8 (15.7%)	
IIB	2 (2.5%)	0	
IIIC1	12 (15.2%)	12 (23.5%)	
IIIC2	1 (1.3%)	0	
Not available	1 (1.3%)	0	
**Histological grade**			0.24 ^c^
G1	9 (11.4%)	6 (11.8%)	
G2	23 (29.1%)	23 (45.1%)	
G3	40 (50.6%)	20 (39.2%)	
Unknown	7 (8.9%)	2 (3.9%)	
**Treatment type**			
Surgery completed	62 (78.5%)	36 (70.6%)	
Surgery stopped due to positive lymph nodes and patients subjected to primary CRT	12 (15.2%)	9 (17.6%)	
Adjuvant CRT after surgery (due to risk factors)	5 (6.3%)	6 (11.8%)	
**Lymph node assessment**			<0.001 ^b^
Lymphadenectomy	12 (15.2%)	29 (56.9%)	
Sentinel lymph node biopsy	67 (84.8%)	22 (43.1%)	
**Follow-up**			0.19 ^d^
Time (months)	30.0 (17.9–47.0)	83.0 (10.0–103.5)	
**Recurrence**			0.53 ^b^
Yes	8 (10.1%)	3 (5.9%)	
No	71 (89.9%)	48 (94.1%)	
**Status at last observation**			
Dead	0	1 (2.0%)	
Alive	79 (100.0%)	50 (98.0%)	
**Overall Survival**			
	30.0 (17.9–47.0)	83.0 (10.0–103.5)	
**Progression-free Survival**			0.36 ^d^
	26.0 (14.3–45.0)	71.3 (5.0–103.5)	

^a^ Mann–Whitney U-test; ^b^ Fisher’s exact test; ^c^ Pearson chi-squared test; ^d^ log-rank test; Eastern Cooperative Oncology Group (ECOG); body mass index (BMI).

**Table 2:** Univariate and multivariate progression-free survival analysis for patients with cervical cancer FIGO ≤ Ib3 undergoing combined versus completely open approach stratified by risk factors.

	Univariate	Multivariate
	Progression-Free Survival Analysis	
Parameter	*p*-Value	*p*-Value
**Age**		
≤65 years vs. >65 years	0.93	1.00
**Histological Type**		
Squamous vs. Other	0.59	0.34
**FIGO Tumor Stage**		
Ia1-Ib1 vs. Ib2-IIIc2	0.15	0.96
**Histological Grade**		
G1 vs. G2+3	0.56	0.70
**Surgical Approach**		
Combined vs. Open	0.37	0.28

## Data Availability

The data presented in this study are available on request from the corresponding author.

## Abbreviations

The following abbreviations are used in this manuscript:

BMI	Body mass index
CRT	Chemoradiotherapy
CT	Computed tomography
ECOG	Eastern Cooperative Oncology Group
ESGO	European Society of Gynaecological Oncology
ESMO	European Society for Medical Oncology
ESP	European Society of Pathology
ESTRO	European Society for Radiotherapy and Oncology
FIGO	International Federation of Gynecology and Obstetrics
HPV	Human papillomavirus
HR	Hazard ratio
ICH	International Council for Harmonisation
IQR	Interquartile range
IRB	Institutional review board
LACC	Laparoscopic approach to cervical cancer
MIS	Minimally invasive surgery
NNT	Number needed to treat
OS	Overall survival
PAP	Papanicolaou (smear)
PET-CT	Positron emission tomography–computed tomography
PFS	Progression-free survival
SLN	Sentinel lymph node
